# Supplementary material for: Genetic Structure of Invasive Baby’s Breath (Gypsophila paniculata L.) Populations in a Michigan Dune System
Source: Plants (Basel). 2020 Aug 31;9(9):1123. doi: 10.3390/plants9091123 (PMC7570141; doi:10.3390/plants9091123)
Supplement: Supplementary file 1 [file plants-09-01123-s001.zip › All_Supplemental_Files/Leimbach-Maus_etal._FigureS1.docx]

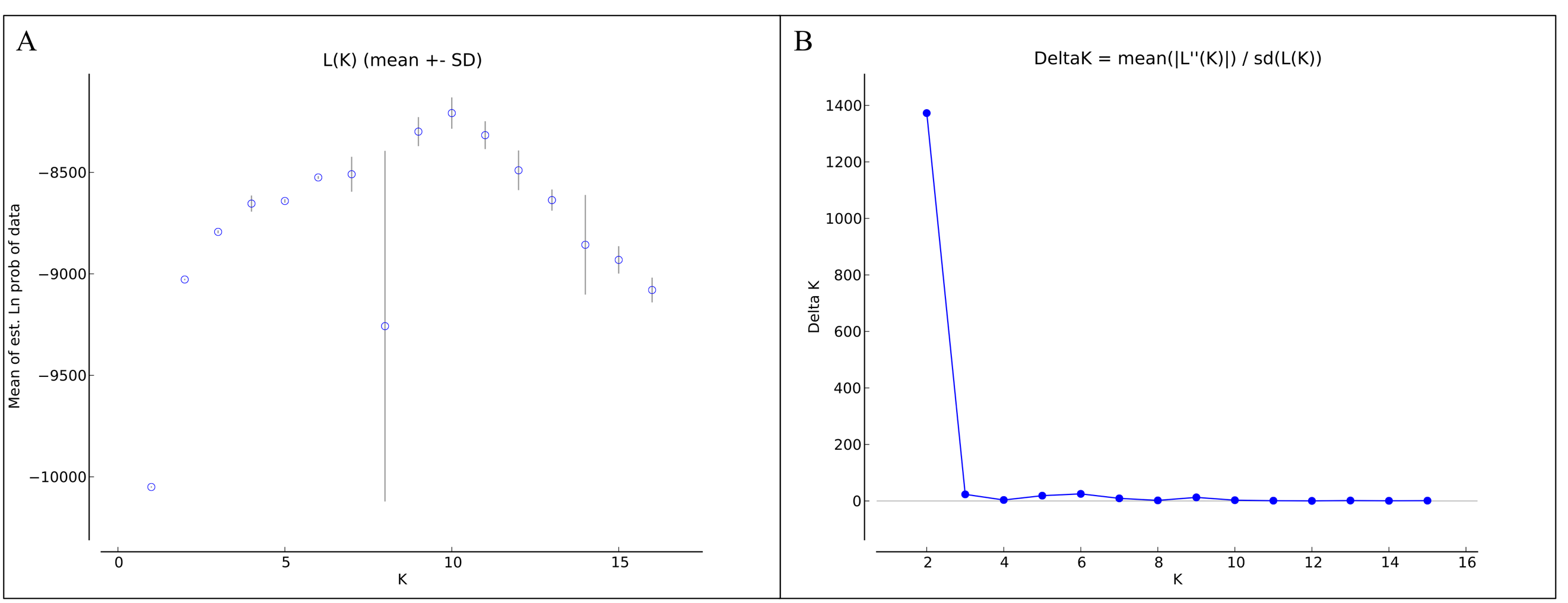


**Figure S1.** Bayesian clustering analysis of all 12 *G. paniculata* populations from the program STRUCTURE (Pritchard et al. 2000). (A) Mean L(K) (± SD) over 10 runs for each value of *K.* (B) Plot of Evanno’s ∆K method (Evanno et al. 2005) where the largest rate of change suggests the highest likelihood of cluster number. This analysis was run without inferring any prior information on sampling location, and two genetic clusters were inferred from this data.
