# Supplementary material for: Genetic Structure of Invasive Baby’s Breath (Gypsophila paniculata L.) Populations in a Michigan Dune System
Source: Plants (Basel). 2020 Aug 31;9(9):1123. doi: 10.3390/plants9091123 (PMC7570141; doi:10.3390/plants9091123)
Supplement: Supplementary file 1 [file plants-09-01123-s001.zip › All_Supplemental_Files/Leimbach-Maus_etal._FigureS3_v2.docx]

**Figure S3.** Minimum spanning network based on Nei’s genetic distance (Nei 1972) matrix of baby’s breath cpSSR data. Created in the *poppr* v2.8.0 package (Kamvar et al. 2014) for R. Illustrates the distribution of haplotypes across the 12 populations. Haplotype size indicates frequency in populations.

Sampling location codes: Grand Marais (GM), Petoskey State Park (PS), Traverse City (TC), Good Harbor Bay (GHB), Sleeping Bear Point (SBP), Dune Climb (DC), Dune Plateau (DP), Empire Bluffs (EB), Platte Bay (PB), South Boundary (SB), Zetterberg Preserve (ZP), Arcadia Dunes (AD).
