# Supplementary material for: Genetic Structure of Invasive Baby’s Breath (Gypsophila paniculata L.) Populations in a Michigan Dune System
Source: Plants (Basel). 2020 Aug 31;9(9):1123. doi: 10.3390/plants9091123 (PMC7570141; doi:10.3390/plants9091123)
Supplement: Supplementary file 1 [file plants-09-01123-s001.zip › All_Supplemental_Files/Leimbach-Maus_etal._FigureS2.docx]

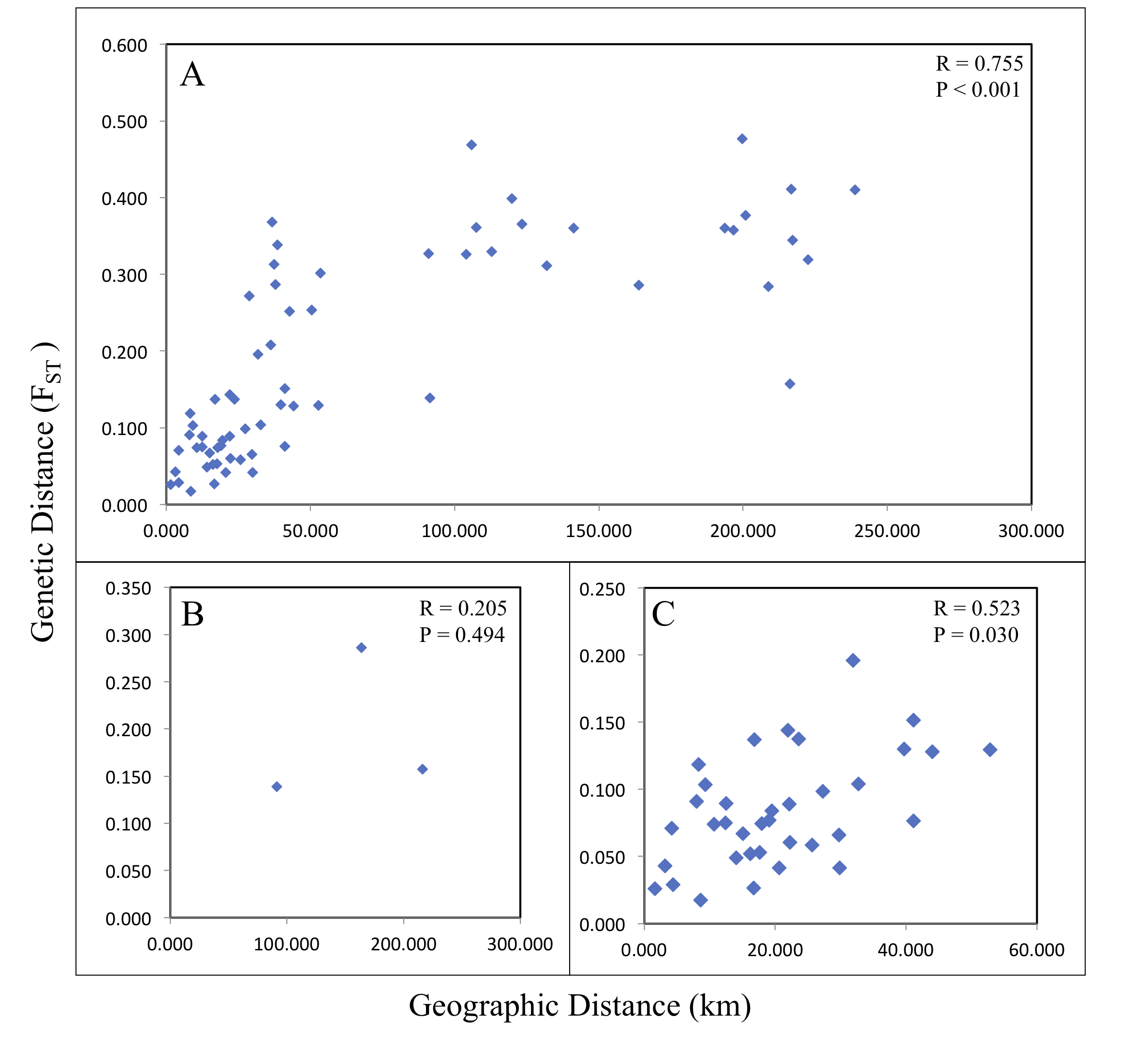


**Figure S2.** Mantel tests using transformed pairwise population F_ST_ values of nSSR data and straight-line distances (km) between populations based on the mean center latitude and longitude of each location. (A) Between all populations, (B) between populations in the northeaster cluster (cluster 1), and (C) between populations in the southwestern cluster (cluster 2) identified from the Bayesian clustering analysis. Reported p-values based on the one-sided alternative hypothesis (H^1^: R > 0).
